# Surgical treatment of chronic acromioclavicular dislocation with biologic graft vs synthetic ligament: a prospective randomized comparative study

**DOI:** 10.1007/s10195-013-0242-2

**Published:** 2013-05-07

**Authors:** Francesco Fauci, Giovanni Merolla, Paolo Paladini, Fabrizio Campi, Giuseppe Porcellini

**Affiliations:** Unit of Shoulder and Elbow Surgery, D. Cervesi Hospital, Via L. Van Beethoven 1, 47841 Cattolica, RN Italy

**Keywords:** Acromioclavicular, Dislocation, Biological, Synthetic, Stabilization

## Abstract

**Background:**

Acromioclavicular (AC) dislocation involves complete loss of articular contact; it is defined as chronic when it follows conservative management or unsuccessful surgical treatment.

**Materials and methods:**

The study compared the clinical and radiographic outcomes of AC joint stabilization performed in 40 patients with chronic dislocation using a biological allograft (group A) or a synthetic ligament (group B). Demographic data included: M/F: 25/15; mean age: 35 ± 3.2 years; previous surgery in 11 patients, including Weaver–Dunn (3), coracoacromial ligament repair (4), stabilization with K-wires (4). Dislocation was type III in 14 (35 %) and type IV in 26 (65 %) patients. Clinical assessment was with the Constant–Murley score (pre- and postoperative) and with the modified UCLA score. Enrollment started in January 2004 and was completed in March 2008. Patients were evaluated at 1 and 4 years. Postoperative X-rays were examined to assess joint stability in the coronal and axial planes, coracoclavicular ossification, and signs of AC joint osteoarthritis and distal clavicular osteolysis.

**Results:**

The “biological” group achieved significantly better clinical scores than the “synthetic” group at both 1 and 4 years. Poor subjective satisfaction and lower clinical scores were found in the 3 patients (1 from group A and 2 from group B) who experienced complete postoperative dislocation. No significant correlations were found with other radiographic parameters.

**Conclusions:**

The biological graft afforded better clinical and radiographic outcomes than the synthetic ligament in patients with chronic AC joint instability. Fixation to the clavicle constitutes the main weakness of both approaches and needs improving.

## Introduction

Acromioclavicular (AC) dislocation involves complete loss of articular contact; a dislocation that is not untreated, is treated conservatively or is treated unsuccessfully by surgery is defined as chronic or inveterate [1, 2]. The AC and coracoclavicular ligaments contribute to anterior–posterior and superior–inferior joint stability, respectively [3]. Complete instability requires rigid fixation of the coracoclavicular ligaments to counteract the AC joint laxity that induces posterior translation of the clavicle. The classification of AC dislocation into 6 degrees of severity, as devised by Rockwood et al. [4], is still the one most commonly used. While there is consensus on the conservative treatment of types I and II, there is still debate over whether types III to V should be managed surgically [5, 6]. Among the surgical approaches developed to treat acute and chronic AC dislocation, some authors [5, 16, 17, 31] have recommended procedures that restore the original joint anatomy and congruity [7]; a number of these techniques use biological or synthetic means [8, 9].

This study compares the clinical and radiographic outcomes of surgical AC joint stabilization performed in 40 patients with chronic dislocation using a biological graft or a synthetic ligament.

## Materials and methods

### Study design

This was a prospective randomized clinical study that was designed to ascertain the results of AC joint stabilization using two systems of fixation. All of the patients gave informed consent prior to being included in the study, which was authorized by the local ethical committee (Cometico AV/IRST no. 4442/C012/I5/169) and was performed in accordance with the Ethical Standards of the 1964 Declaration of Helsinki as revised in 2000.

### Randomization and sample size

The intent-to-treat population included 40 patients who were fully randomized using a block list that was generated by dedicated software (Research Randomizer, version 3.0, 2011). Envelopes containing the treatment assignments were used to randomize the patients in the two groups. A power analysis was performed in which a 7-point difference in the Constant score between the two groups was required, and a standard deviation of 6 points. Using these parameters, it was calculated that a minimum of 38 subjects were needed.

### Study population

**Fig. 1:**
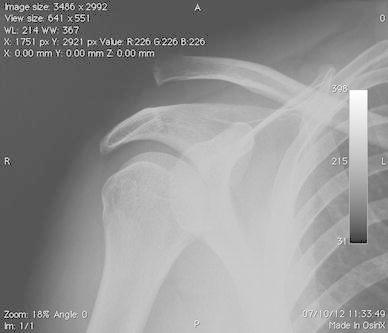
Complete AC joint dislocation, right shoulder (type IV of Rockwood et al. [4])

Enrollment started in January 2004 and was completed in March 2008. Forty consecutive patients with chronic AC joint dislocation who underwent surgical stabilization using a biological graft (group A) or a synthetic ligament (group B) were enrolled. The two groups were age- and sex-matched; their demographic data are reported in Table 1. Inclusion criteria were complete dislocation (Fig. 1) of type III or greater according to Rockwood et al. [4]; age <60 years; ≥100 % dislocation of the AC joint surface; pain at rest and during activity; loss of strength in overhead movements; failure of previous conservative (>6 months) or surgical treatment; absence of sequelae from scapular trauma, rotator cuff tear, and glenohumeral instability. The interval from trauma to surgery was 16 months (range 4–22) in group A and 12 months (range 5–26) in group B. Patients were randomly assigned to one of the two treatments.

**Table 1:** Demographic data for the patients enrolled in the study

Variable	Group A	Group B	*p* value
No. of patients	20	20	0.5624
Gender (M/F)	15/5	10/10	0.0638
Mean age (years ± SD)	36 ± 4.3	34 ± 2.8	0.6297
Dominant arm (right/left) (%)	13 (65)/7(35)	11 (55)/9(45)	0.6498
Overhead workers (N°) (%)	12 (60)	10 (50)	0.4361
Previous surgery (N°) (%)	8 (40)	3 (15)	0.0541
Degree of dislocation			
Type III	8 (40)	6 (30)	0.8173
Type IV	12 (60)	14 (70)	0.7382

### Clinical assessment

Eleven patients (27.5 %) had undergone surgical treatment at other institutions as follows: Weaver–Dunn procedure, 3 patients (5 %, 1 in group A and 2 in group B); coracoacromial ligament repair with non-absorbable suture, 4 patients (10 %, 2 in group A, 2 in group B); stabilization with K wires, 4 patients (all group B). All patients complained of pain involving the AC joint and the trapezius that worsened with cross-arm adduction. Active ROM was full in all patients. Weakness beyond 90° of elevation was seen in 5 patients (12.5 %, 2 in group A and 3 in group B). Patients were examined for keloids, AC joint deformity, pain on palpation or during passive mobilization in forward elevation and forced adduction, and joint instability during active mobilization. The Constant–Murley score [10] was used for clinical assessments before and after the operation and the modified UCLA score [11] was employed after the operation. This study required that clinical follow-up was performed at 1 and 4 years.

### Radiographic evaluation

AP and axillary views were examined to assess AC joint stability in the coronal and axial planes, coracoclavicular ossification, signs of osteoarthritis, and distal clavicular osteolysis. X-rays were routinely performed at 2 months; for the requirements of the current study, additional radiograms were taken at 1 and 4 years. Postoperative AC joint stability was assessed according to Rosenorm and Pedersen [12]; the AC joint was considered to be stable if it showed no dislocation compared to the contralateral joint; subluxated if the dislocation was ≤50 % of the contralateral joint; or dislocated if there was complete dislocation accounting for ≥100 % of the AC joint surface.

Coracoclavicular ossification was deemed incomplete if there was no continuity between clavicle and coracoid process, and complete if it obliterated the coracoclavicular space.

Arthritis was considered to be present if the joint showed joint space narrowing, osteophytes, or sclerosis. Clavicular osteolysis was defined as signs of demineralization around the screws or on the lateral portion of the clavicle.

### Statistical analysis

Clinical scores were expressed as the mean ± standard deviation. Student’s unpaired *t* test was applied to assess differences between the two groups. Significance was set at 5 % (*p* < 0.05).

### Surgical technique

**Fig. 2:**
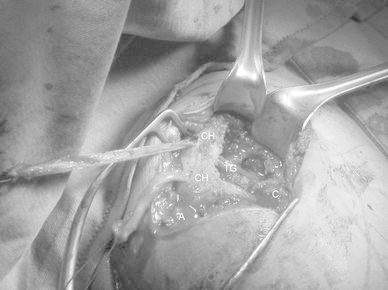
Intraoperative image showing the biological graft as it is being passed under the base of the coracoid and through the holes in the clavicle after AC joint reduction. *C* coracoid, *CH* clavicular holes, *TG* tendon graft, *A* acromion

**Fig. 3:**
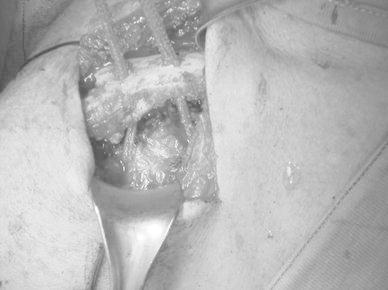
Intraoperative image showing the synthetic ligament (LARS LAC^®^, Arc sur Tille, France) as it is being passed under the coracoid and through the clavicular holes

The operations were performed in the beach chair position using a superior approach from the AC joint to the tip of the coracoid. First, the distal end of the clavicle and the acromion were exposed to remove the interposed fibrocartilaginous meniscus and about 1 cm of bone on the distal end of the clavicle. The coracoid was dissected free of adhesions to pass the graft under its base (Figs. 2, 3). The sites of the two clavicular holes were determined in the frontal plane by following the anatomical insertion of the coracoclavicular ligaments: the conoid ligament, which is found approximately 4.5 cm from the lateral border of the clavicle, and the trapezoid tubercle, which lies 2.5 cm from it. The lateral hole (“trapezoid ligament tunnel”) should be located about 2 cm from the margin of the AC joint and the medial hole (“conoid ligament tunnel”) approximately 4.5 cm from the lateral margin of the AC joint (Fig. 2). The directions of the two tunnels should be slightly convergent. Group A patients received a semitendinosus graft (Rizzoli Orthopaedic Institute, Bologna, Italy) (Fig. 2) that was fixed to the clavicle with polylactic acid screws (Arthrex^®^, Naples, FL, USA) 4.5–10 mm in diameter and 5.7–15 mm in length. Group B patients were treated with a synthetic ligament (LARS LAC^®^, Arc sur Tille, France) 20 mm in diameter that was fixed to the clavicle with titanium screws 4.7–5.7 mm in diameter and 15 mm in length. In group A, the lateral stump of the graft was fixed to the acromion using transosseous sutures in order to reproduce the anatomy and serve the function of the capsular ligaments in controlling anteroposterior joint stability. The ligament was passed through the holes using suture thread to hold its extremities; the clavicle was reduced ensuring that its distal end was aligned with the acromion in both the coronal and axial planes. Finally, the wound was sutured in layers. The arm was immobilized in a sling for 30 days, passive mobilization was begun after 1 month, and active exercise in a water pool at 40 days. Strength exercises were allowed at 75 days.

## Results

### Clinical outcomes

Group A: the mean Constant–Murley score increased more than doubled from 43.5 ± 6.1 to 88 ± 10 at 1 year (*p* = 0.0097) and to 94.2 ± 4.9 at 4 years (*p* = 0.0093). The mean UCLA score was 17.8 ± 1.8 at 1 year and 18.2 ± 1.7 at 4 years (Table 2).

**Table 2:** Preoperative and postoperative clinical scores

Follow-up	Group A	Group B	*p* value
Constant–Murley score			
Preoperative	43.5 ± 6.1	44.05 ± 8.9	–
1 year	88 ± 10	59 ± 7.9	0.0092
4 years	94.2 ± 4.9	85.9 ± 16	0.0626
Modified UCLA score			
1 year	17.8 ± 1.8	11.8 ± 4.9	–
4 years	18.2 ± 1.7	15.4 ± 4.2	–
Subjective satisfaction			
Preoperative	8.7 ± 3.4	8.4 ± 2.6	–
1 year	3.7 ± 1.6	4.1 ± 1.5	0.2782
4 years	3.9 ± 1.8	3.9 ± 1.4	0.7935

Data refer to mean + standard deviation

Group B: the mean Constant–Murley score rose from 44.05 ± 8.9 to 59 ± 7.9 at 1 year (*p* = 0.0049) and to 85.9 ± 16 at 4 years (*p* = 0.0089). The mean UCLA score was 11.8 ± 4.9 at 1 year and 15.4 ± 4.2 at 4 years (Table 2).

Subjective satisfaction was good in 17 patients (85 %) from group A and in 14 patients (55 %) from group B. A significant improvement was registered for both groups at 1 year (*p* = 0.011 and at 4 years (*p* = 0.014). None of the 40 patients had to change their habits after the operation due to the clinical outcome, including returning to sports or a job.

We did not find a significant difference between the 11 patients previously treated surgically and the study population in their clinical scores and subjective satisfaction.

### Radiographic findings

#### Group A

**Fig. 4:**
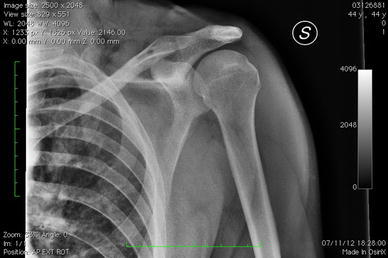
Postoperative X-rays: left AC joint stabilized with the biological graft

**Fig. 5:**
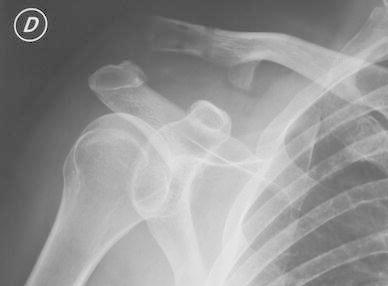
Postoperative X-rays: complete dislocation after stabilization with the biological graft. Note the coracoclavicular ossification

**Fig. 6:**
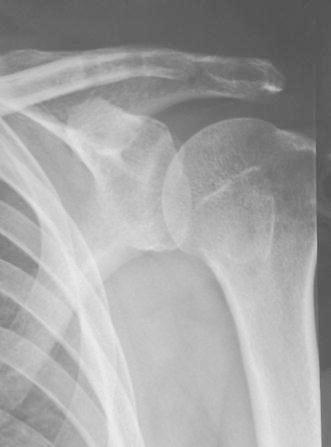
Clavicular osteolysis around the screws in a stable AC joint treated with the biological graft

The AP and axillary X-ray views taken immediately after the operation showed a stable AC joint in 19 patients (95 %) (Fig. 4). At 2 months, 1 shoulder had subluxated and another showed complete dislocation (Fig. 5). At 1 year, subluxation with posterior translation of the clavicle <50 % of the articular surface was seen in 4 patients (20 %). There were no additional cases of instability at 4 years (Fig. 5). Incomplete coracoid ossification was found only at 1 year (5 shoulders, 25 %). AC joint arthritis was seen in 4 patients (20 %) at 1 year and in 8 additional patients (40 %) at 4 years. Osteolysis around the screws and on the distal end of the clavicle was found in 5 shoulders (20 %) at 1 year and in 13 shoulders (65 %) at 4 years (Fig. 6). Osteolysis was not found in the control radiograms at 2 months. The radiographic findings of this group are reported in Table 3. The 3 patients with X-ray evidence of joint subluxation also had osteolysis around the screws. The radiographic findings of this group are reported in Table 3.

**Table 3:** Postoperative radiographic findings

Follow-up	X-ray findings			
	2 months	1 year	4 years	Total
Group A				
Subluxation	1	3	0	4
Complete dislocation	1	0	0	1
AC joint arthritis	0	4	8	12
Coracoclavicular ossification	0	5	0	5
Clavicular osteolysis	0	5	13	18
Group B				
Subluxation	1	3	0	4
Complete dislocation	1	1	0	2
AC joint arthritis	0	11	2	13
Coracoclavicular ossification	0	7	1	8
Clavicular osteolysis	2	16	2	20

#### Group B

**Fig. 7:**
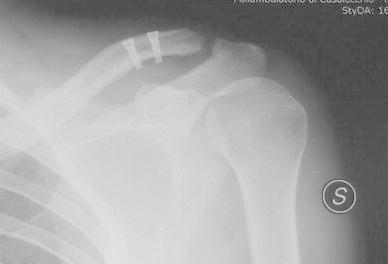
Postoperative X-rays: left AC joint stabilized with the synthetic ligament (LARS LAC^®^)

**Fig. 8:**
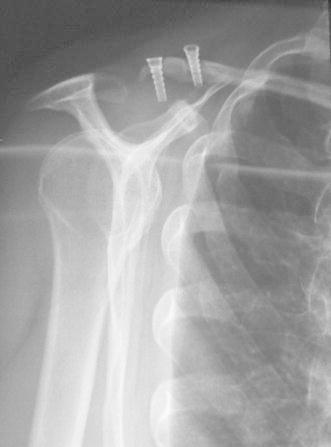
Distal clavicular fracture, osteolysis and screw loosening in a patient treated with the synthetic ligament (LARS LAC^®^)

The postoperative radiograms in the AP and axillary views showed a stable AC joint in 12 shoulders (60 %) (Fig. 7). Complete dislocation was found in 2 patients (10 %) at 2 months and at 1 year; no additional cases were found at 4 years. In 1 patient, loosening of the lateral screw, fracture of the distal end of the clavicle, and incomplete rupture of the synthetic ligament (Fig. 8) required removal of the ligament and stabilization using coracoacromial ligament transposition according to a modified Weaver–Dunn procedure [13]. Six patients (30 %) with subluxation that was seen in the AP X-ray view had <50 % posterior translation of the of the articular surface of the clavicle. Incomplete coracoid ossification was found in 7 patients (35 %) at 1 year and in another (5 %) at 4 years. An arthritic joint was found in 11 patients (55 %) at 1 year and in 2 additional patients (10 %) at 4 years. Osteolysis around the screws was seen in 2 shoulders (10 %) at 2 months and in 16 shoulders (80 %) at 1 year (Fig. 8). At 4 years, all patients had asymptomatic clavicular osteolysis. The radiographic findings of this group are reported in Table 3.

### Clinical–radiographic correlations

Subjective satisfaction was not related to the degree of AC joint reduction. Although early postoperative radiographs showed partial loss of AC joint alignment in 3 patients (15 %) from group A and in 6 (30 %) from group B, poor satisfaction was only reported by 4 group B patients (20 %).

At 4 years, <50 % partial dislocation (subluxation), which was found in 4 patients from group A (20 %) and in 4 from group B (20 %), did not correlate with the clinical scores (*p* > 0.05). Poor subjective satisfaction and lower clinical scores were found in the 3 patients (1 from group A and 2 from group B) who had experienced complete joint dislocation after the operation. No correlation was found between clinical score and coracoclavicular ossification, clavicular osteolysis, or AC joint osteoarthritis.

## Discussion

From 1861 [14] to the present, 60 different surgical procedures have been devised to treat acute and chronic AC joint dislocation, but finding the gold standard has proved an elusive task. In 1972, Weaver–Dunn [15] proposed the transposition of the coracoacromial ligament to the lateral portion of the clavicle. This approach involves sacrificing the coracoacromial ligament (a humeral stabilizer). The interest in this type of technique, which is based on the assumption that AC joint reduction and anatomical restoration provide more satisfactory outcomes [16], has recently been revived by the introduction of synthetic ligaments [17, 18] and biological grafts [16, 19]. Techniques based on the transposition of the patient’s tendons that show resistance to cyclic loading, similiar to rigid osteosynthesis (screws, plates, pins, metal or synthetic cerclage) [20, 21] but with lower rates of intra- and postoperative complications, were developed to address these problems [8, 19, 22–24]. Bailey [25] was the first to report the results of tendon transposition; Dewar and Barrington [26] used only coracoid transposition and obtained better mid-term outcomes compared with the Weaver–Dunn procedure in young patients [27]. Although transposition of the coracoid with the conjoint tendon reinforces the reconstructed coracoacromial ligament, it involves a greater risk of coracoid fracture and musculocutaneous nerve injury; furthermore, bone cerclage may result in coracoid or clavicle osteolysis.

Materials that are used for artificial ligaments include polyester, Dacron^®^, Dupont^®^, Wilmington^®^, Notthingam^®^ [8, 23], carbon fiber [28], polytetrafluoroethylene (Gore-Tex^®^) [29], and PET (LARS LAC^®^) [24]. The characteristic interwoven fibers and the porosity of the synthetic ligament promote fibroblast colonization and make the ligament biocompatible and resistant to traction and torsion; nonetheless, intolerance, inflammation, and rejection have been described [30]. Tendon autografts or allografts were initially used in salvage procedures after failed coracoacromial ligament reconstruction [19]. The most widely used allografts are semitendinosus [19], gracilis, hallux extensor [31], and peroneus brevis tendons [18]. Biocompatibility, resistance, and rigidity of the system used for joint reduction are crucial for postoperative stability in chronic AC joint dislocation.

Although good outcomes of synthetic [21] and biological grafts [16] have been (separately) described in several reports, no single study has, to our knowledge, used both materials and compared them. Although anatomical AC joint reconstruction cannot restore original stability to the joint, tendon grafts provide greater resistance and rigidity than the Weaver–Dunn procedure [29]. Analysis of the results of our study disclosed significantly greater clinical scores in the “biological” compared with the “synthetic” group at both follow-up time points, with mean intergroup differences in Constant–Murley score of >29 points at 1 year and >8.9 points at 4 years, and mean differences in modified UCLA score of 6 points at 1 year and 2.8 points at 4 years.

Eleven out of 40 patients were previously surgically treated using different surgical techniques, which affected the articular biomechanics of the AC joint in different ways, and consequently influenced the homogeneity of the study population. In these patients, we found a higher incidence of periarticular ossifications, clavicular osteolysis, and fibrous adhesions intraoperatively, which made it more difficult to expose the clavicle and acromion. Furthermore, the passage of the graft under the coracoid required a longer surgical step due to the thickening of the surrounding soft tissues. Despite these difficulties, we did not find any significant effects on the clinical scores and AC joint stability based on the X-rays for this subgroup of patients.

Our clinical findings are consistent with the aforementioned case-series studies describing the use of synthetic or biologic grafts. Specifically, Carofino et al. [16] reported a significant difference between preoperative and postoperative clinical scores when using a semitendinosus allograft. On the other hand, Morrison et al. [21] reported satisfactory early and midterm outcomes using a synthetic graft. Postoperative radiographic assessment showed three complete AC joint dislocations that negatively affected the clinical scores, while subluxations were only associated with poor subjective satisfaction in 20 % of group B patients. None of the remaining radiographic measures investigated correlated with clinical outcomes. Coracoclavicular ossification is usually related to surgical exposure of the coracoclavicular space [32], but it is unclear how its onset, site, and extension affects clinical outcomes. Although the incidence of clavicular osteolysis is greater in patients managed surgically than in those managed conservatively [33], this has been related to the biomechanical effects of AC injury rather than to the surgical procedure per se [32]. In this study, patients with clavicular osteolysis were more numerous in the “synthetic” group; in this group, the sizes of the osteolytic areas increased in 90 % of the patients, and the only patient with dislocation complicated by clavicular fracture was treated with the LARS LAC^®^ ligament. The number of osteolytic areas and their sizes in our 40 patients were not related to loss of postoperative AC alignment, consistent with other reports [34, 35]. AC joint stability is not related to poor clinical outcome [36], whereas clavicle malrotation or anteposition may contribute to arthritic changes [21, 36]. Comparison of our patient groups showed a greater rate of osteoarthritis in the “synthetic” than in the “biological” group (80 vs 40 %), with no significant correlations with clinical scores or X-ray evidence of instability found for either group. A number of considerations can be drawn from these findings:

(i) Postoperative AC joint stability is the main factor affecting final outcome; the best results were recorded in patients with completely stable joints.
(ii) Although the synthetic graft is effective from a biomechanical standpoint, graft shredding and wear and bone remodeling around the screws can compromise mechanical strength over time, particularly in elderly patients and in those with poor clavicle bone thickness or osteoporosis.
(iii) Biological grafts provide joint stability in the axial and the coronal planes through suture of the lateral stump of the graft to the acromion, a finding confirmed by recent [16] and earlier [3] studies; axial stability appears more difficult to restore using a synthetic graft.
(iv) Biological grafts are fixed to the clavicle with resorbable screws and are a valuable option when treating patients with postoperative recurrence of dislocation due to synthetic graft failure.

The major limitations of this study are the small sample size, the lack of inter- and intraobserver data, and the absence of patients treated with tendon autografts.

In conclusion, our findings show that biological grafts provide biocompatible, durable, and effective reduction, as well as better clinical outcomes and radiographic findings than synthetic ligaments, and thus represent the most reasonable alternative to the Weaver–Dunn [15] procedure in shoulders with chronic AC joint instability. Graft fixation to the clavicle is the major weakness of both procedures and should be improved.
